# Meniscus body position and its change over four years in asymptomatic adults: a cohort study using data from the Osteoarthritis Initiative (OAI)

**DOI:** 10.1186/1471-2474-15-32

**Published:** 2014-02-05

**Authors:** Katharina Bruns, Fredrik Svensson, Aleksandra Turkiewicz, Wolfgang Wirth, Ali Guermazi, Felix Eckstein, Martin Englund

**Affiliations:** 1 Institute of Anatomy and Musculoskeletal Research, Paracelsus Medical University, Salzburg, Austria; 2 Department of Orthopedics, Clinical Sciences Lund, Lund University, Lund, Sweden; 3 Chondrometrics GmbH, Ainring, Germany; 4 Department of Radiology, Boston University School of Medicine, Boston, MA, USA; 5 Clinical Epidemiology Research & Training Unit, Boston University School of Medicine, Boston, MA, USA

**Keywords:** Knee, Meniscus, Magnetic resonance imaging, Osteoarthritis

## Abstract

**Background:**

A high degree of meniscal body extrusion on knee magnetic resonance imaging has been shown to be strongly associated with development of knee osteoarthritis. However, very little is known about meniscal position in the asymptomatic knee and its natural history. Hence our objective was to study meniscal body position and its change over 4 years in asymptomatic adults.

**Methods:**

Cohort study using data from the Osteoarthritis Initiative (OAI) involving four clinical sites in the United States (Baltimore, Maryland, Pawtucket, Rhode Island, Columbus, Ohio, and Pittsburgh, Pennsylvania). We studied both knees from 118 subjects (mean age 55 years, 61% women, mean body mass index 24.4) from the OAI “non-exposed” reference cohort free of knee pain, radiographic knee osteoarthritis and risk factors for knee osteoarthritis at baseline. We assessed mid-coronal intermediate-weighted 3-Tesla magnetic resonance images from baseline and the 2- and 4-year follow-up visit. One observer measured tibia plateau, meniscal body width and meniscal body extrusion in both compartments. We calculated meniscal overlap distance on the tibial plateau, % coverage, and extrusion index compared to tibia width. Potential trends in position over the 4-year period were evaluated using a linear mixed-effects regression model.

**Results:**

The mean (SD) values at baseline for medial meniscal body extrusion and overlap distance were 1.64 mm (0.92) and 10.1 mm (3.5), and coverage was 34.4% (11.9). The corresponding values for the lateral compartment were 0.63 mm (0.73), 9.8 mm (2.4), and 31.0% (7.7). Medial meniscus body extrusion index was greater in female knees (p = 0.03). There was slight increase in medial meniscal body extrusion over 4 years (0.040 mm/year [95% CI: 0.019-0.062]). The other variables were relatively stable.

**Conclusions:**

In asymptomatic adults, the relative degree of meniscus body extrusion is more pronounced in female knees. Although a slight increase in extrusion over time was noted for the medial body, positions were relatively stable within subjects over time.

## Background

The meniscus is a fibrocartilaginous structure between femur and tibia with the important task of load distribution but potentially also shock absorption [1-3]. About 70% of the load passes through the medial tibiofemoral compartment and 30% through the lateral compartment in normally aligned knees [4]. While there is limited biomechanical studies of effects of different meniscus position *per se*, there is evidence that a displaced meniscus, e.g., due to a root tear, will no longer provide optimal load transmission in the knee, thus result in increased cartilage contact stress in similar manner as after partial or total meniscectomy [5,6].

Substantial extrusion of the meniscal body, i.e., when the peripheral part of the mid portion of the meniscus is markedly located outside the tibial joint margin, is considered a structural feature that is strongly related to the incidence and progression of knee osteoarthritis (OA) [7-9]. A high degree of meniscal body extrusion on knee magnetic resonance (MR) imaging has been shown to be strongly associated with development of radiographic knee OA, cartilage loss, and the development of ipsilateral bone marrow lesions [10-12]. Potentially it may also be causing knee symptoms, e.g., by affecting the often pain sensitive knee capsule and synovial tissue [13]. Meniscal extrusion has also been reported to be associated with meniscal degeneration and extensive tears [14-16]. However, when discussing pathological meniscal position, one first needs to know more about meniscal position in the asymptomatic knee. For instance, medial meniscal extrusion of up to 3 millimetres (mm) is common in persons 40 years of age or older without knee symptoms [7]. Still, there is limited data published on this topic and also knee sizes and the sizes of menisci may vary considerably [17,18]. Hence, the relative degree of meniscal extrusion may be very different between subjects even if the absolute measure of extrusion is identical. Also, to the best of our knowledge there are no studies of meniscal position over time in individuals free of knee OA.

Therefore, in this study we aimed to gain insight on medial and lateral meniscal body position (cross-sectional analysis) and its potential change (longitudinal analysis) over a period of 4 years in asymptomatic persons without risk factors and radiographic knee OA at baseline. We used a 2-dimensional quantitative measurement technique originally reported by Hunter et al. [12] Our main objectives were to answer the following questions:

a) What is meniscus body position like in knees from asymptomatic adults in a midcoronal image slice? As meniscal tears are common in asymptomatic persons [19], but associated with altered meniscus position [14,15], we aimed to present data both collapsed and stratified by meniscal tear.

b) Further, are there any trends in body position detectable over a 4-year time-period? We hypothesized that meniscus body position would remain reasonably stationary in asymptomatic subjects in this relatively intermediate time period.

## Methods

### Study sample

Data used in the preparation of this article were obtained from the Osteoarthritis Initiative (OAI) database, which is available for public access at http://www.oai.ucsf.edu/. Specific dataset used were 0.F.2, 3.F.1 and 6.F.1, i.e., the “non-exposed” reference cohort of the OAI. This cohort consists of 122 subjects (47 men and 75 women; age range: 45-79 years) who were invited to an annual exam with knee MR imaging. Inclusion criteria were:

● No pain, aching or stiffness in either knee in the past year;

● No radiographic OA in the tibiofemoral joint of either knee in the site readings performed during recruitment;

● No eligibility risk factors for knee OA present with the exception of age ≥ 70 years.

In brief, the exclusion factors were: certain knee symptoms associated with OA in the past 12 months, overweight, history of knee injury and/or knee surgery, family history of total knee replacement, Heberden’s nodes in both hands and repetitive knee bending.

**Figure 1:**
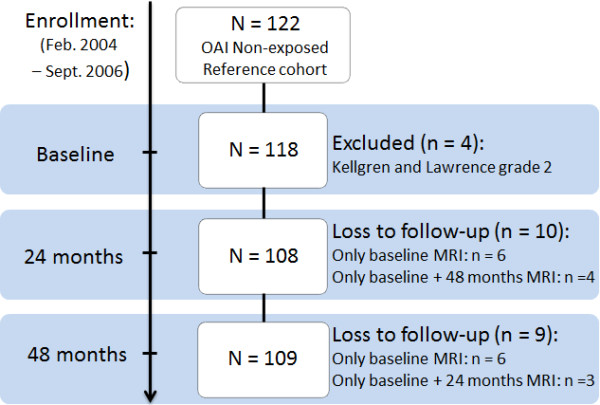
Flow chart detailing the study sample.

Four individuals had to be excluded due to presence of radiographic tibiofemoral OA (Kellgren & Lawrence grade 2) that retrospectively was noted in the central readings. Seventeen knees with KLG 1 in the central readings were included in the analysis. Hence, for our cross-sectional analyses (using baseline data) the study sample consisted of 118 subjects. For longitudinal analyses, using MR imaging data from the baseline exam, 2-year follow-up and 4-year follow-up, 6 subjects were lost to follow-up, and additional 3 subjects only appeared for baseline and the 2-year follow up, and another 4 subjects only appeared for baseline and the 4-year follow-up (Figure 1).

The OAI was approved by the respective institutional review boards for the University of California, San Francisco and the four OAI clinical centres (University of Pittsburgh, Ohio State University, University of Maryland, Baltimore, Memorial Hospital of Rhode Island). All patients gave informed consent to participate in this study.

### MR imaging protocol and measurements

Siemens 3 Tesla MR imaging scanners, one at each of the 4 clinical study centres located in the United States were used to take baseline (enrolment period 2004 to 2006) and annually follow-up scans of both left and right knees over a period of 4 years.

**Figure 2:**
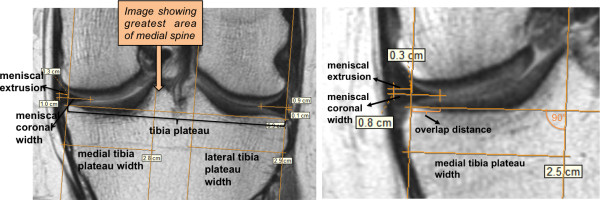
Example of measurements on mid-coronal 3 T intermediate weighted knee MR images using eFilm 3.4 software (the vertical lines are supporting lines perpendicular to the tibial plateau created to aid measurements).

One observer (KB) trained by an orthopaedic surgeon (FS), who was blinded to subject characteristics but had knowledge of time sequence (no image fusion was performed for longitudinal measurements), measured both left and right knees on the longitudinal (baseline, 2-year and 4-year) coronal intermediate-weighted turbo spin-echo (IW TSE) MR images (repetitive time = 3,700 ms; echo time = 29 ms; slice thickness = 3 mm and in-plane resolution 0.37 × 0.46 mm). For this study purpose, we refer “mid-coronal” to the single slice presenting the greatest area of the medial spine, which was the slice chosen for the measurements. If this was difficult to differentiate (2 slices had similar area of the spine), we picked the image which showed the greatest width of the tibia plateau. The observer measured tibia plateau width (from the edge of the tibial plateau, there were no osteophytes), medial and lateral tibia plateau width, medial and lateral meniscal coronal width, and meniscal body extrusion to the closest mm using eFilm 3.4 software (Figure 2) [12]. Fifty randomly picked knees from the baseline examination were remeasured. Intra-observer reliability (intra-class correlation coefficient) for the parameters ranged from 0.70 to 0.99. We further tested if there were any systematic differences in rotation between repeat MRI examinations in a random subset of 11 knees (by counting slices from the posterior tibial margin to the (our) mid coronal slice).

One clinical investigator (ME), with background in orthopedics and experienced in reading meniscal status, studied all right and left knee MR images at the baseline exam for the presence of medial or lateral meniscal tears or destruction/maceration in the anterior horn, body, or posterior horn using the IW TSE sagittal and coronal fat-suppressed sequences. The reader regarded an increased meniscal signal as indicative of a meniscal tear when it communicated with the inferior, superior, or free edge of the meniscal surface (or more than one of those) on at least two consecutive images (or for a radial tear, if it was visible on both the coronal and sagittal images) [20].

We used anatomic (femorotibial) axis alignment data in OAI based on the posterioanterior knee radiographs done with the knee in fixed flexion using the Synaflexer frame [21]. The measurement of anatomic axis alignment involves the definition of the femoral axis using a coordinate system based on the shape of the femoral condyles that is defined as part of the location specific joint space width measurement methods used by Dr. Duryea [22]. The tibial axis is defined by the direction and center of the tibial shaft at a distance 10 cm below the tibial plateau. There is an offset from the anatomic axis to the mechanical axis because the shaft of the femur does not include the femoral neck which takes off medially from the femoral shaft. In order to classify knees into varus and valgus, we used a sex neutral offset of +4 degrees that has been reported to have the highest kappa values in terms of agreement between anatomic and mechanical alignments [23]. Hence, we based our categorization considering 2 or more degrees of valgus (mechanical axis) as valgus, 2 degrees or more of varus (mechanical axis) as varus and everything in between as neutral. These cut-offs corresponded to an anatomic axis > = − 2 degrees as valgus, and < = − 6 degrees as varus.

### Statistics

We calculated the meniscal body extrusion index as: [meniscus body extrusion]/[tibia width]*100, i.e., taking into account differences in knee size, often seen between e.g., women and men but also within the sexes. The corresponding values were also reported after dividing by ipsilateral tibia plateau width instead of tibia width. Further, we calculated overlap distance between the free (inner) edge of the meniscal body and the margin of the tibial plateau as: [meniscus body width]-[meniscus body extrusion]. Finally, we calculated the proportion (%) of the width of the ipsilateral tibial plateau covered by meniscus as: ([meniscus body width]-[meniscus body extrusion])/[ipsilateral tibia plateau width].

We also present data stratified by meniscal tear, i.e., evaluating subjects *with* and *without* meniscal tear at the baseline exam, separately, because tears are reported to be associated with more meniscal extrusion [15]. To evaluate potential differences between baseline parameters in unpaired groups, we used a 2-level linear mixed regression model with a patient as a random effect to control for the correlation of measurements made in the same patient with adjustment for age and sex. Potential changes in meniscal body extrusion, overlap distance, or meniscal body coverage over the 4-year time period (3 time points) were analysed using a linear 3-level mixed effects regression model to account for correlation between measurements of two knees in the same person. The only fixed effect was time (using actual number of days between MR images obtained). We performed a sensitivity analysis for the longitudinal analyses stratifying by knees which were free of knee pain, aching or stiffness also at the 2-year and 4-year follow-up and those that were not. We considered a two-tailed p-value of 0.05 or less as statistically significant (SPSS software version 19, and STATA 12).

## Results

The mean (SD) age of study subjects, n = 118 (46 men and 72 women) was 55.0 (7.5) years (Table 1). At the baseline exam, 17 of 236 medial menisci (7.2%) and 9 of 236 lateral menisci (3.8%) in 26 of 236 knees (11.0%) in 24 subjects (20.3%) were noted to have relatively minor non-displaced meniscal tears (typically degenerative horizontal cleavages, flap tears or radial tears located to the body and/or posterior horn). Two subjects had minor meniscal maceration/destruction of the meniscal body free edge (one subject in both the medial and the lateral compartment and one in the lateral compartment only).

**Table 1:** Characteristics of the study subjects

**Characteristic**	**N = 118**
Women, n (%)	72 (61)
Age, mean ± SD (range) *years*	55.0 ± 7.5 (45–78)
Body mass index, mean ± SD (range) *kg/m*^*2*^	24.4 ± 3.2 (18–34)
Kellgren and Lawrence grade, n (%)*	
0	99 (85)
1	17 (15)
Medial meniscal tear, n (%)	
Right knee	9 (7.6)
Left knee	8 (6.8)
Lateral meniscal tear, n (%)	
Right knee	6 (5.1)
Left knee	3 (2.5)
Anatomic axis alignment*†	
Right knee, mean ± SD degrees	−5.5 ± 1.6
Varus, n (%)	41 (35)
Neutral, n (%)	74 (64)
Valgus, n (%)	1 (1)
Left knee, mean ± SD degrees	−4.9 ± 1.6
Varus, n (%)	28 (24)
Neutral, n (%)	84 (72)
Valgus, n (%)	4 (3)

*Missing data for 2 subjects.

†We considered the femorotibial (anatomic) axis alignment < = −6.0° as varus and > = −2.0° as valgus.

### Meniscal body extrusion (baseline)

The mean (SD) absolute measure of medial body extrusion (average of right and left knees) was 1.64 (0.92) mm. The corresponding values for the lateral compartment were 0.63 (0.73) mm. The mean (SD) extrusion index (relative to tibia width) for the medial meniscus body was 2.3 (1.3) and for the lateral meniscus body 0.9 (1.0) (Table 2).

**Table 2:** Meniscal measurements and calculated variables from the baseline exam. All values are means (SD) if not otherwise stated

				**Ipsilateral meniscal tear (in the same compartment)**			
	**Total sample**			**No**		**Yes**	
	**Both knees**	**Right knee**	**Left knee**	**Right knee**	**Left knee**	**Right knee**	**Left knee**
*Medial meniscus body*	N = 236	N = 118	N = 118	N = 109	N = 110	N = 9	N = 8
Width, mm	11.8	12.0 (3.4)	11.6 (3.3)	12.0 (3.4)	11.7(3.3)	11.0 (3.1)	11.0 (2.7)
Extrusion, mm	1.6	1.9 (1.0)	1.4 (0.77)	1.9 (1.0)	1.4 (0.73)	1.9 (0.78)	2.1 (0.99)
Extrusion index*	2.3	2.6 (1.4)	2.0 (1.1)	2.6 (1.4)	1.9 (1.0)	2.5 (1.1)	2.7 (1.3)
Extrusion index (ipsilat)†	5.7	6.4 (3.5)	4.9 (2.8)	6.4 (3.6)	4.8 (2.7)	6.2 (2.8)	7.0 (3.5)
Overlap distance§, mm	10.1	10.1 (3.6)	10.2 (3.5)	10.2 (3.6)	10.3 (3.5)	9.1 (3.1)	8.9 (3.3)
Coverage‡, %	34.4%	34.3 (12.0)%	34.5 (11.8)%	34.7 (12.2)%	34.9 (11.8)%	29.2 (9.7)%	28.7 (10.7)%
*Lateral meniscus body*	N = 236	N = 118	N = 118	N = 112	N = 115	N = 6	N = 3
Width, mm	10.4	10.4 (2.3)	10.3 (2.6)	10.5 (2.3)	10.4 (2.5)	9.0 (1.9)	7.7 (2.1)
Extrusion, mm	0.63	0.64 (0.79)	0.61 (0.67)	0.62 (0.77)	0.61 (0.67)	1.00 (1.10)	0.67 (0.58)
Extrusion index*	0.9	0.9 (1.1)	0.8 (0.9)	0.9 (1.1)	0.8 (0.9)	1.4 (1.6)	1.0 (0.8)
Extrusion index (ipsilat)†	2.0	2.1 (2.6)	1.9 (2.1)	2.0 (2.5)	1.9 (2.1)	3.2 (3.7)	2.3 (2.0)
Overlap distance§	9.8	9.8 (2.3)	9.7 (2.5)	9.9 (2.3)	9.8 (2.5)	8.0 (1.4)	7.0 (2.6)
Coverage‡	31.1%	31.3 (7.4)%	30.8 (7.9)%	31.6 (7.4)%	31.0 (7.8)%	26.2 (7.6)%	23.3 (7.8)%

*[meniscal body extrusion]/[tibia width] * 100.

†[meniscal body extrusion]/[ipsilateral tibia plateau width] * 100.

§[meniscal coronal width] – [meniscal body extrusion].

‡([meniscal coronal width] – [meniscal body extrusion])/[ipsilateral tibia plateau width].

**Figure 3:**
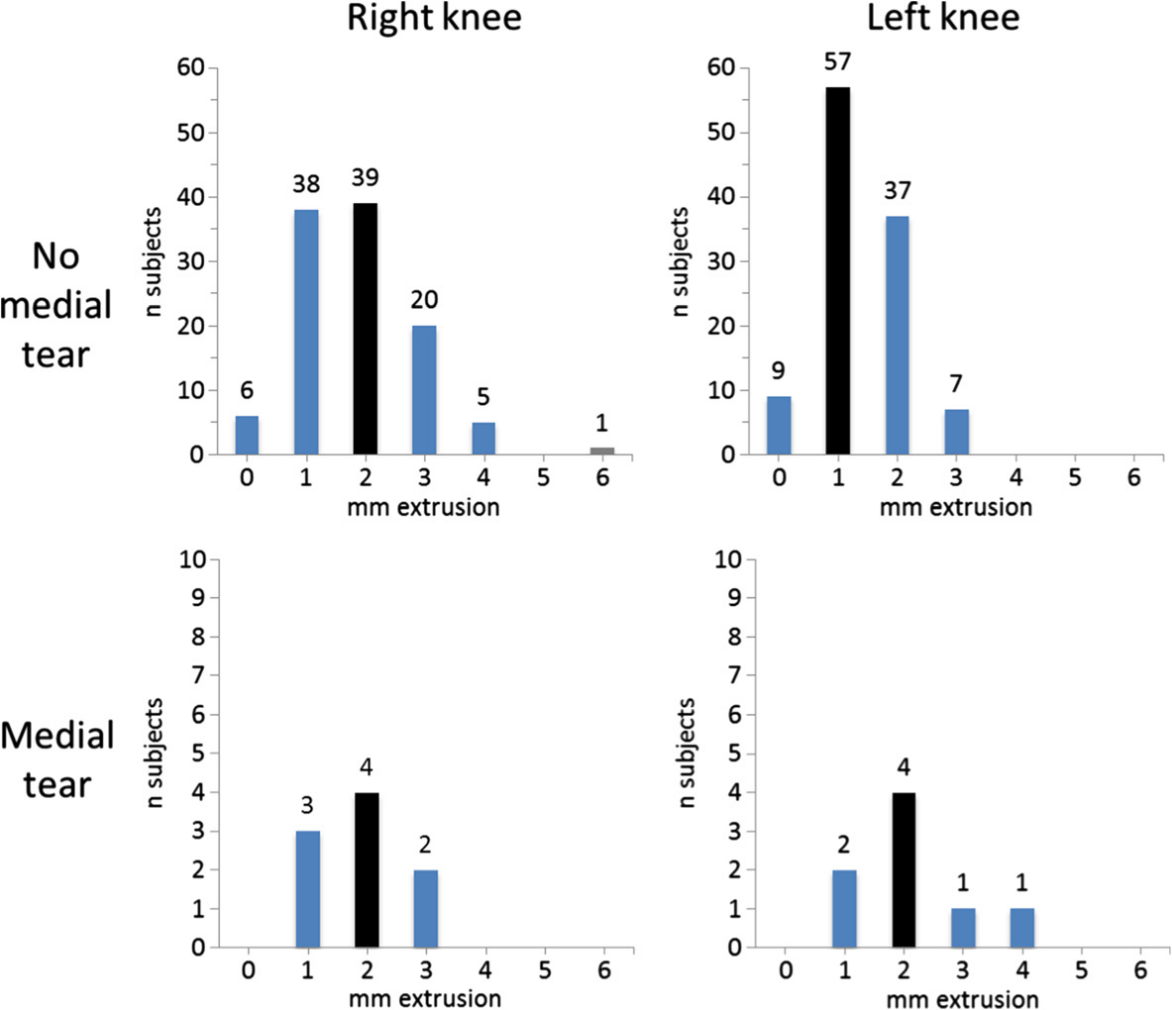
**Frequencies of medial meniscal body extrusion at baseline in the study sample of 118 subjects without radiographic osteoarthritis (OA) and without risk factors for knee OA.** Black bars illustrate the median value.

**Figure 4:**
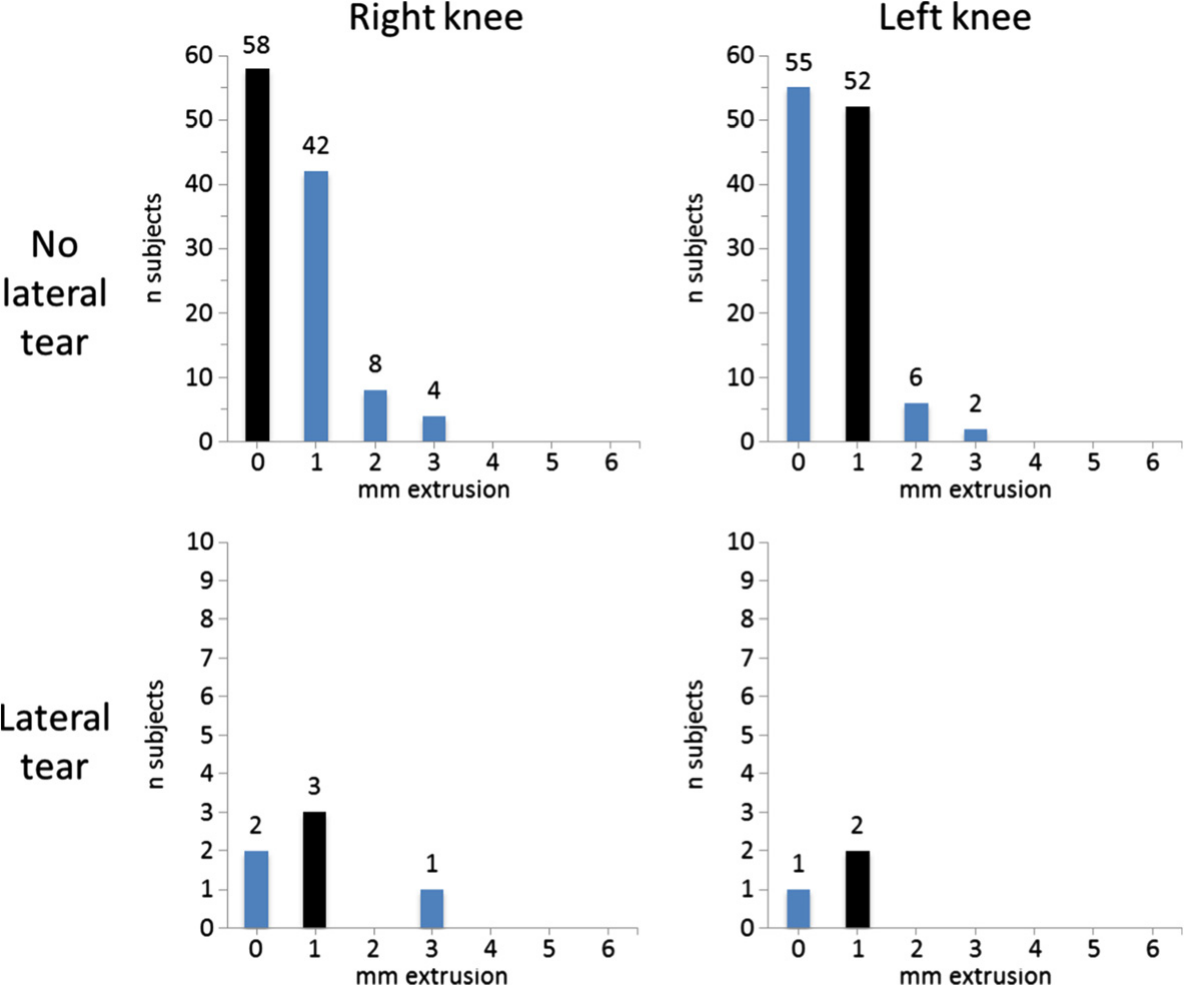
**Frequencies of lateral meniscal body extrusion at baseline in the study sample of 118 subjects without radiographic osteoarthritis (OA) and without risk factors for knee OA.** Black bars illustrate the median value.

We found knees with medial meniscal tear to have a tendency to more medial meniscal body extrusion than knees with intact medial menisci (mean [SD] 2.0 [0.87] mm vs. 1.62 [0.92] mm; p = 0.05) (Figure 3). The corresponding values for lateral meniscal body extrusion were 0.89 (0.93) mm in those with tear vs. 0.62 (0.72) mm in those without lateral tear (p = 0.31) (Figure 4).

Men had a mean (SD) medial meniscal body extrusion at baseline of 1.60 (1.10) mm, and women 1.67 (0.79) mm (p = 0.49). Laterally, the corresponding absolute values of meniscal extrusion were 0.70 (0.77) mm vs. 0.58 (0.71) mm (p = 0.31), respectively. Since female knees are often smaller than male ones (mean [SD] tibia width: 69.23 [2.96] mm vs. 78.87 [3.48] mm; p < 0.001), and size of knees within persons within the same sex also vary greatly, we related the amount of extrusion to tibia plateau width and to ipsilateral tibia plateau width. Medially, women had a mean (SD) extrusion index of 2.4 (1.2) and men of 2.0 (1.4) (p = 0.03) related to total tibia plateau width. Laterally, the mean (SD) ratios were very similar: 0.8 (1.0) vs. 0.9 (1.0), respectively (p = 0.75). The corresponding index when relating to the ipsilateral compartment width for medial side were 6.0 (2.9) vs. 5.1 (3.6) for women and men (p = 0.059), and 2.0 (2.4) vs. 2.0 (2.3) (p = 0.87) for the lateral side.

### Meniscal body overlap distance and coverage (baseline)

The overlap distance of the central part of the medial meniscal body was on average (SD) 10.14 (3.53) mm. The corresponding results from the lateral compartment showed a mean (SD) overlap distance of 9.76 (2.42) mm.

Stratified by the presence of medial meniscal tear we found a mean (SD) medial overlap distance of 9.00 (3.12) mm in the knees with medial tear vs. a mean of 10.22 (3.55) mm in knees without medial tear, respectively (p = 0.31). In the lateral compartment, we found a mean (SD) overlap distance of 7.67 (1.80) mm in knees with lateral meniscal tear, while those without lateral tear showed a mean overlap distance (SD) of 9.84 (2.40) mm (p < 0.001). The central part of the medial meniscal body covered on average (SD) 34.4% (11.9) of the medial tibia plateau width. The corresponding result for the lateral compartment was 31.0% (7.7).

**Figure 5:**
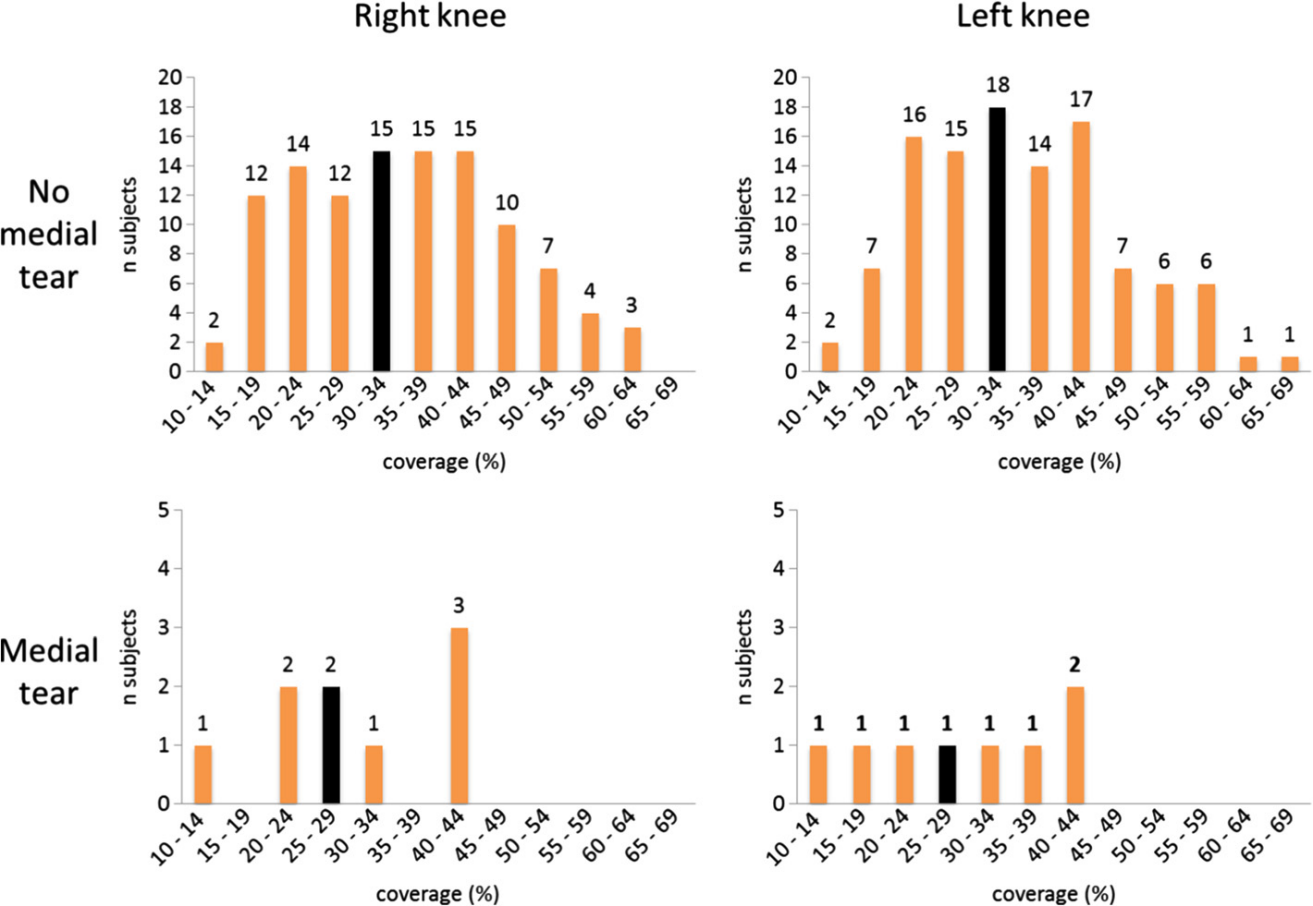
**Frequency of medial coverage (% of ipsilateral tibia plateau width).** Black bars illustrate the median value.

**Figure 6:**
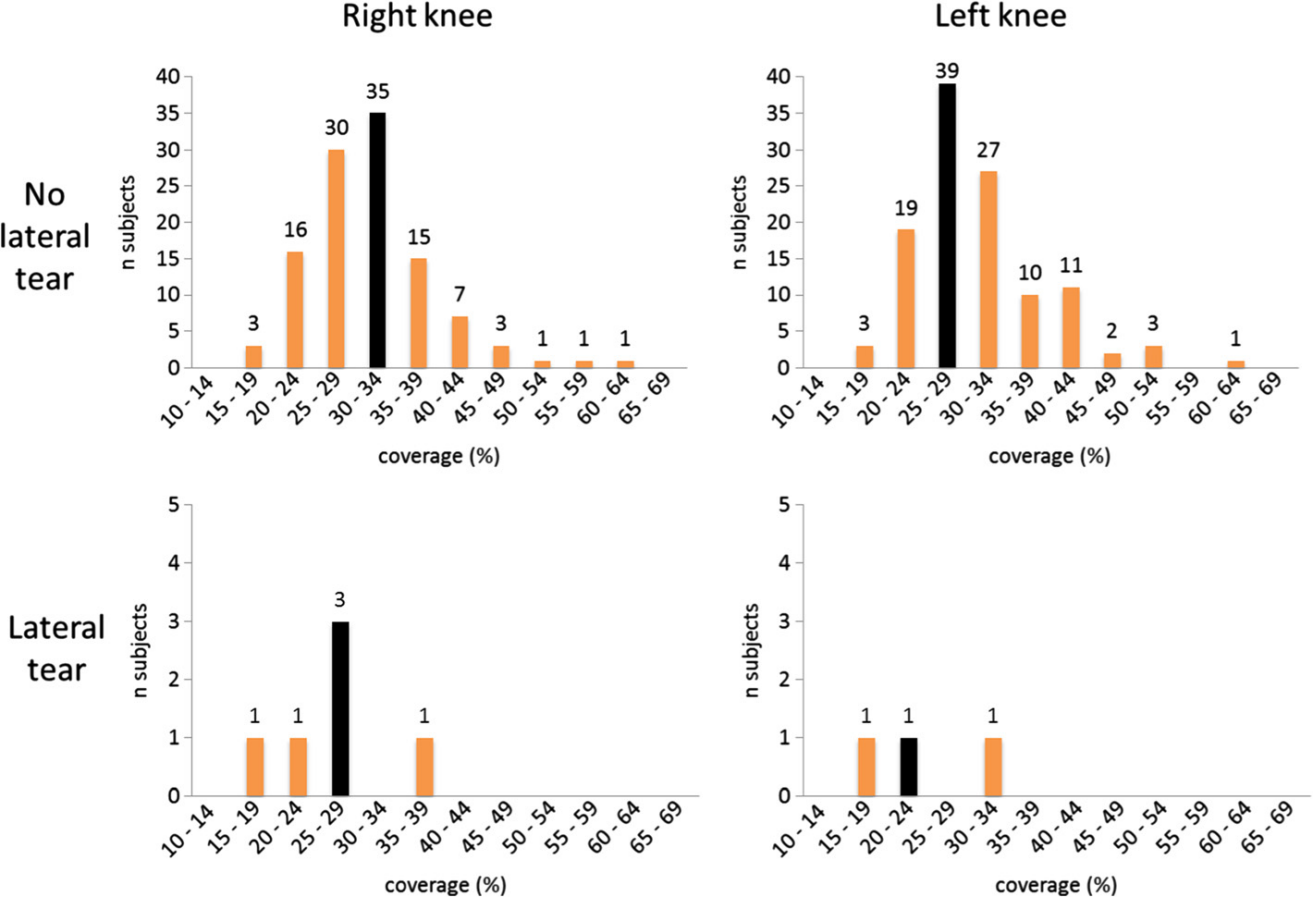
**Frequency of lateral coverage (% of ipsilateral tibia plateau width).** Black bars illustrate the median value.

Stratified by the presence of medial meniscal tear, we found a mean (SD) medial coverage of 29.0% (9.9) in the knees with medial tear vs. 34.8% (11.9) in knees without medial tear (p = 0.29) (Figure 5). In the lateral compartment we found a mean (SD) coverage of 25.2% (7.3) in knees with lateral meniscal tear, while those without lateral tear had a coverage of 31.3% (7.6) (p < 0.001) (Figure 6).

Medially in men, the meniscus covered on average 33.4% (11.9) of the ipsilateral tibia plateau width and 35.1% (11.9) in women (p = 0.6). The corresponding figures for the lateral compartment in men and women were 30.3% (7.6) and 31.5% (7.7), respectively (p = 0.3).

### Change over time in body extrusion, overlap distance, and coverage

In the 2- and 4-year follow up, measurements for the medial side suggested a trend for increase in meniscal body extrusion over time, with an annually increase of 0.040 mm (95% CI: 0.019 – 0.062). The result was statistically significant, in both women and men, when stratifying the analysis by sex (data not shown). The analysis for meniscal overlap distance in the medial compartment suggested no essential change, -0.002 mm (95% CI: -0.072 – 0.069) per year. Further, no change in medial coverage was detected during the 4-year interval: -0.00012% (95% CI: -0.002 – 0.002), i.e., the percentage of the ipsilateral tibia plateau that is covered by meniscus stayed medially at about 34% (Table 3).

**Table 3:** Mean change in meniscus measures from baseline to the 4-year follow-up in asymptomatic subjects without knee osteoarthritis (OA) and without risk factors for knee OA

		**Change from baseline to 4-year follow-up**		
	**N**	**Extrusion (mm)**	**Overlap distance (mm)**	**Coverage (%)**
*No meniscal tear at baseline (ipsilateral)*				
Medial body, mean change (SD)				
Right knee	101	0.27 (0.76)	−0.20 (2.36)	−0.5 (8.0)
Left knee	94	0.06 (0.60)	0.26 (1.98)	0.7 (7.0)
Lateral body, mean change (SD)				
Right knee	103	0.00 (0.54)	0.23 (1.46)	0.7 (4.8)
Left knee	98	0.12 (0.75)	0.26 (1.96)	0.7 (6.1)
*Meniscal tear at baseline (ipsilateral)*				
Medial body, mean change (SD)				
Right knee	8	0.25 (0.89)	−0.25 (2.49)	−1.0 (8.2)
Left knee	7	−0.14 (1.22)	−0.43 (1.99)	−1.4 (6.3)
Lateral body, mean change (SD)				
Right knee	6	0.17 (1.17)	0.50 (0.84)	1.6 (3.3)
Left knee	3	0.33 (0.58)	−0.33 (1.53)	−1.5 (4.0)

The results for the lateral side did not show any statistically significant trends in extrusion over time, mean 0.011 mm (95% CI: -0.007-0.029) per year (Table 3). However, the results for overlap distance on the lateral side suggested a mean increase of 0.069 mm (95% CI: 0.019 – 0.119) per year. Analysis for lateral coverage also suggested no essential changes although it was statistically significant: 0.002% (95% CI: 0.0004-0.004) (Table 3).

In the sensitivity analysis, stratifying by knees that were free of knee pain, aching or stiffness also at the 2-year and 4-year follow-up (n = 189) and those that were not (n = 47), the results for change in meniscal body extrusion, overlap distance, and coverage remained essentially the same for both compartments in both stratum (data not shown).

We found no evidence of systematic rotation of follow-up MR images. In most (18 of 21; one time point missing) of the follow-up exams, the mid coronal slice occurred as the same slice number as the baseline exam, and no slice was deviating more than +/−1 from the baseline exam.

## Discussion

We have provided reference values for medial and lateral meniscal body position in knees of asymptomatic persons without radiographic knee OA and without common risk factors for knee OA using a two-dimensional measurement technique on coronal MR images. Interestingly, we found that there was a small tendency to increased medial meniscal body extrusion over a 4-year period. However, most meniscal position parameters remained relatively stable. We also provide data for a relative measure of meniscal extrusion, i.e., which takes into account the fact that knee size (and size of menisci) may vary between subjects and an absolute measure of extrusion may not give justice to these size differences.

Since physiological degradation of meniscal tissue and its displacement may be a slow process, there is a need of more sensitive measuring methods of meniscal position than semi-quantitative scoring, which is incorporated in the most commonly used knee MR imaging scoring systems of knee OA [24,25]. For instance, in subjects with knee OA or with risk factors for knee OA, within-grade changes in semi-quantitative MR imaging assessment are valid and may increase sensitivity in detecting longitudinal changes of cartilage and bone marrow lesions [26]. It is plausible that this is true also for meniscal changes. Full segmentation of meniscal volume and its relation to the tibial plateau can provide very detailed information but is time consuming, hence challenging for larger sample sizes or in daily clinical practice [27-29].

To our knowledge, there is not much written about the physiological meniscal position in the asymptomatic knee free of radiographic OA [30] and in particular its potential change over time. Several earlier studies distinguished between “minor” and “major” extrusion and in radiologic literature, it seems to have become common to define a threshold of 3 mm or more on coronal images as “pathological” extrusion [14]. Consequently, an extrusion of the meniscus body less than 3 mm is often regarded as of less clinical importance. This value is supported by results for meniscal extrusion measurements in a control group of asymptomatic persons, which however included subjects with evidence of radiographic OA changes [7]. The informative value about pathology of a “minor” extrusion is ambiguous. In contrast to the above-mentioned 3 mm cut-off is the 2 and 5 mm separation, which is used in the semi-quantitative MR imaging scoring systems BLOKS and MOAKS [24,25]. Instead of an absolute measure, we would like to advocate for the use of a relative measure, similar to the early work addressing meniscal extrusion as seen on MR imaging [8]. In the report by Kenny, the ratio was constructed by dividing by the meniscus width. However, we propose to rather use tibia width (or ipsilateral tibia plateau width) in the coronal plane because meniscal body width may be affected by degeneration, tear or meniscus surgery. Thus, we are currently addressing this question in knees with and without radiographic OA to more accurately determine the most suitable measure to divide by and to define cut-offs for clinical and research use. Interestingly, in relative terms women tended to have more medial meniscal extrusion at the baseline exam compared to men, which also has been previously described by Bloecker et al. [18]. This tendency in our study was not evident unless taking into account knee size, i.e., using our proposed extrusion index construct. This finding may be important as knee OA is more common in women, and meniscus position and collateral ligament laxity may play important roles in pathogenesis.

Our finding of more medial meniscal extrusion in knees with medial meniscal tear is in agreement with previous cross-sectional reports, suggesting that tears are associated with increased meniscal extrusion [14-16]. Knees with meniscal tears in our study also tended to have less tibial plateau coverage compared to knees without tears. Between 40% and 70% of load has been reported to be transmitted by the menisci; the rest through direct contact of articular cartilage [31]. Being exposed to this great stress, its position (and coverage) plays an often critical role in maintaining a healthy knee. Our measurements suggest a mean coverage of 34% of width medially and of 31% laterally. While the lateral meniscus coverage is the same as suggested for patients with symptomatic knee OA, the medial coverage is 13% greater in asymptomatic knees compared to the results of symptomatic OA knees using the same measurement technique [12]. The wider range of coverage in sample of asymptomatic individuals most likely reflects the variety of anatomical shapes and dimensions of the tibial plateau and meniscus body [17,18].

While meniscal coverage diminishes in OA affected subjects due to meniscal destruction and radial displacement [12], the coverage in asymptomatic knees at baseline stayed about the same during a 4-year time period, both medially and laterally. The tendency in the present study to increased extrusion of the medial meniscus body with time, not affecting coverage and overlap distance to the same extent, might potentially indicate increased bulging of the peripheral meniscal margin (more convex shape), i.e., not so much radial displacement (shift in position). Of note, meniscal extrusion is a combined construct of radial displacement and potential change in meniscus width, e.g., due to bulging of the peripheral meniscus margin and/or meniscus hypertrophy [32,33]. The clinical relevance of our longitudinal findings is unknown, and is a question for forthcoming studies.

This study has important limitations that we would like to point out. Due to funding limitations and time constraints our aims included only the study of meniscal body position. Hence, the study of potential anterior and posterior horn movements is a question for future work. The number of knees with meniscal tear is small to provide robust estimates of association. Since knee alignment have great impact on biomechanical force transmission which may influence the degree of extrusion, a larger sample allowing enough numbers in different alignment categories would be valuable in future work [34]. For this study, we used a relatively straightforward two-dimensional quantitative measurement technique on the mid-coronal slice, i.e., a technique that does not consume as much time or needs specialized software as full volume segmentation, and therefore is more suitable for clinical practice. However, it does not provide as much detailed information as techniques doing full segmentation of the meniscus body [27-29]. The reading software we used (Efilm 3.4) only allowed measurements to the closest millimetre. As we are dealing with small measurements such as meniscal extrusion, measurements to a tenth of a millimetre would have been preferable using alternate imaging software. Our calculated coverage represents the % width of the ipsilateral tibia plateau that is covered by meniscus in the mid-coronal MRI slice. Although this variable is not representing the whole area of the meniscus covering the plateau, we suggest it likely provides a fairly representative proxy of the cartilage coverage of the meniscal body. Finally, the reader being familiar with the time sequence in which the images were taken, sensitivity to eventual changes in size and position was increased but potential bias cannot be excluded [35]. Any change in rotation between exams or misclassification of mid-coronal slice is expected to be non-differential which will only bias findings towards the null.

## Conclusions

Using 3-Tesla MR imaging data from the OAI “unexposed” reference cohort, we provide data of meniscus medial and lateral body position in asymptomatic subjects including a new relative measure of extrusion as a ratio of extrusion by the tibia width. These measures may be useful tools in future studies of meniscus position in OA knees and for clinical practice. Further, our findings suggest that over a 4-year time frame there is on average a small increased medial body extrusion (mainly due to increased bulging) even in asymptomatic middle-aged or elderly subjects without knee OA at baseline. The association of these alterations with incidence of cartilage changes, radiographic OA etc. is a topic for further research as well as potential meniscal changes in position in younger adults.

## Competing interests

Wolfgang Wirth has a part-time appointment with Chondrometrics GmbH, a company providing MR image analysis services, and is co-owner of Chondrometrics GmbH. Ali Guermazi is President and co-founder of the Boston Imaging Core Lab (BICL), a company providing radiological reading services to academic researchers and to industry. He provides consulting services to Genzyme. Felix Eckstein is CEO and co-owner of Chondrometrics GmbH. He provides consulting services to MerckSerono, Novartis, Sanofi Aventis, Perceptive, Bioclinica and Abbot.

## Authors’ contributions

Conception and design: ME, FE. Acquisition of data: KB, FS, ME. Analysis and interpretation of the data: KB, FS, AT, WW, AG, FE, ME. Drafting of article: KB, FS. Reviewing for important intellectual content: AT, WW, AG, FE, ME. Final approval of submitted version: KB, FS, AT, WW, AG, FE, ME. Obtaining of funding: KB, ME.

## Pre-publication history

The pre-publication history for this paper can be accessed here:

http://www.biomedcentral.com/1471-2474/15/32/prepub

## References

[B1] Kurosawa H Fukubayashi T Nakajima H Load-bearing mode of the knee joint: physical behavior of the knee joint with or without menisci Clin Orthop Relat Res 1980 149 283 290 7408313

[B2] Walker PS Erkman MJ The role of the menisci in force transmission across the knee Clin Orthop Relat Res 1975 109 184 192 1173360 10.1097/00003086-197506000-00027

[B3] Andrews S Shrive N Ronsky J The shocking truth about meniscus J Biomech 2011 44 16 2737 2740 10.1016/j.jbiomech.2011.08.026 21924725

[B4] Hsu RW Himeno S Coventry MB Chao EY Normal axial alignment of the lower extremity and load-bearing distribution at the knee Clin Orthop Relat Res 1990 255 215 227 2347155

[B5] Harner CD Mauro CS Lesniak BP Romanowski JR Biomechanical consequences of a tear of the posterior root of the medial meniscus. Surgical technique J Bone Joint Surg Am 2009 91 Suppl 2 257 270 19805589 10.2106/JBJS.I.00500

[B6] Bae JY Park KS Seon JK Kwak DS Jeon I Song EK Biomechanical analysis of the effects of medial meniscectomy on degenerative osteoarthritis Med Biol Eng Comput 2012 50 1 53 60 10.1007/s11517-011-0840-1 22038241

[B7] Gale DR Chaisson CE Totterman SM Schwartz RK Gale ME Felson D Meniscal subluxation: association with osteoarthritis and joint space narrowing Osteoarthritis Cartilage 1999 7 6 526 532 10.1053/joca.1999.0256 10558850

[B8] Kenny C Radial displacement of the medial meniscus and Fairbank’s signs Clin Orthop Relat Res 1997 339 163 173 9186215 10.1097/00003086-199706000-00022

[B9] Sugita T Kawamata T Ohnuma M Yoshizumi Y Sato K Radial displacement of the medial meniscus in varus osteoarthritis of the knee Clin Orthop Relat Res 2001 387 171 177 11400879 10.1097/00003086-200106000-00023

[B10] Englund M Guermazi A Roemer FW Aliabadi P Yang M Lewis CE Torner J Nevitt MC Sack B Felson DT Meniscal tear in knees without surgery and the development of radiographic osteoarthritis among middle-aged and elderly persons: the Multicenter Osteoarthritis Study Arthritis Rheum 2009 60 3 831 839 10.1002/art.24383 19248082 PMC2758243

[B11] Englund M Guermazi A Roemer FW Yang M Zhang Y Nevitt MC Lynch JA Lewis CE Torner J Felson DT Meniscal pathology on MRI increases the risk for both incident and enlarging subchondral bone marrow lesions of the knee: the MOST study Ann Rheum Dis 2010 69 10 1796 1802 10.1136/ard.2009.121681 20421344 PMC2966967

[B12] Hunter DJ Zhang YQ Niu JB Tu X Amin S Clancy M Guermazi A Grigorian M Gale D Felson DT The association of meniscal pathologic changes with cartilage loss in symptomatic knee osteoarthritis Arthritis Rheumatism 2006 54 3 795 801 10.1002/art.21724 16508930

[B13] Wenger A Englund M Wirth W Hudelmaier M Kwoh K Eckstein F Investigators OAI Relationship of 3D meniscal morphology and position with knee pain in subjects with knee osteoarthritis: a pilot study Eur Radiol 2012 22 1 211 220 10.1007/s00330-011-2234-z 21842432

[B14] Costa CR Morrison WB Carrino JA Medial meniscus extrusion on knee MRI: is extent associated with severity of degeneration or type of tear? AJR Am J Roentgenol 2004 183 1 17 23 15208101 10.2214/ajr.183.1.1830017

[B15] Allen DM Crema MD Marra MD Guermazi A Wyman BT Hellio Le Graverand MP Englund M Brandt KL Hunter DJ The relationship between meniscal tears and meniscal positioning Ther Adv Musculoskel Dis 2010 2 6 315 323 10.1177/1759720X10383198 PMC3383493 22870457

[B16] Crema MD Roemer FW Felson DT Englund M Wang K Jarraya M Nevitt MC Marra MD Torner JC Lewis CE Factors associated with meniscal extrusion in knees with or at risk for osteoarthritis: the multicenter osteoarthritis study Radiology 2012 264 2 494 503 10.1148/radiol.12110986 22653191 PMC3401352

[B17] Stone KR Freyer A Turek T Walgenbach AW Wadhwa S Crues J Meniscal sizing based on gender, height, and weight Arthroscopy 2007 23 5 503 508 10.1016/j.arthro.2006.12.025 17478281

[B18] Bloecker K Englund M Wirth W Hudelmaier M Burgkart R Frobell RB Eckstein F Revision 1 size and position of the healthy meniscus, and its correlation with sex, height, weight, and bone area- a cross-sectional study BMC Musculoskelet Disord 2011 12 248 10.1186/1471-2474-12-248 22035074 PMC3215228

[B19] Englund M Guermazi A Gale D Hunter DJ Aliabadi P Clancy M Felson DT Incidental meniscal findings on knee MRI in middle-aged and elderly persons N Engl J Med 2008 359 11 1108 1115 10.1056/NEJMoa0800777 18784100 PMC2897006

[B20] De Smet AA Tuite MJ Use of the “two-slice-touch” rule for the MRI diagnosis of meniscal tears AJR Am J Roentgenol 2006 187 4 911 914 16985134 10.2214/AJR.05.1354

[B21] Nevitt MC Peterfy C Guermazi A Felson DT Duryea J Woodworth T Chen H Kwoh K Harris TB Longitudinal performance evaluation and validation of fixed-flexion radiography of the knee for detection of joint space loss Arthritis Rheumatism 2007 56 5 1512 1520 10.1002/art.22557 17469126

[B22] Iranpour-Boroujeni T Li J Lynch J Nevitt M Duryea J A new method to measure anatomic knee alignment: a tool for large studies of OA? Osteoarthr Cartil 2012 20 Suppl 1 S20 10.1016/j.joca.2014.06.011 25278076

[B23] Felson DT Cooke TD Niu J Goggins J Choi J Yu J Nevitt MC Group OAII Can anatomic alignment measured from a knee radiograph substitute for mechanical alignment from full limb films? Osteoarthr Cartil 2009 17 11 1448 1452 10.1016/j.joca.2009.05.012 19505430 PMC2763977

[B24] Hunter DJ Guermazi A Lo GH Grainger AJ Conaghan PG Boudreau RM Roemer FW Evolution of semi-quantitative whole joint assessment of knee OA: MOAKS (MRI Osteoarthritis Knee Score) Osteoarthr Cartil 2011 19 8 990 1002 10.1016/j.joca.2011.05.004 21645627 PMC4058435

[B25] Hunter DJ Lo GH Gale D Grainger AJ Guermazi A Conaghan PG The reliability of a new scoring system for knee osteoarthritis MRI and the validity of bone marrow lesion assessment: BLOKS (Boston Leeds Osteoarthritis Knee Score) Ann Rheum Dis 2008 67 2 206 211 10.1136/ard.2006.066183 17472995

[B26] Roemer FW Nevitt MC Felson DT Niu J Lynch JA Crema MD Lewis CE Torner J Guermazi A Predictive validity of within-grade scoring of longitudinal changes of MRI-based cartilage morphology and bone marrow lesion assessment in the tibio-femoral joint–the MOST study Osteoarthr Cartil 2012 20 11 1391 1398 10.1016/j.joca.2012.07.012 22846715 PMC3863692

[B27] Stone KR Stoller DW Irving SG Elmquist C Gildengorin G 3D MRI volume sizing of knee meniscus cartilage Arthroscopy 1994 10 6 641 644 10.1016/S0749-8063(05)80062-3 7880356

[B28] Wirth W Frobell RB Souza RB Li X Wyman BT Le Graverand MP Link TM Majumdar S Eckstein F A three-dimensional quantitative method to measure meniscus shape, position, and signal intensity using MR images: a pilot study and preliminary results in knee osteoarthritis Magn Reson Med 2010 63 5 1162 1171 10.1002/mrm.22380 20432287

[B29] Bowers ME Tung GA Fleming BC Crisco JJ Rey J Quantification of meniscal volume by segmentation of 3 T magnetic resonance images J Biomech 2007 40 12 2811 2815 10.1016/j.jbiomech.2007.01.016 17391677 PMC2084402

[B30] Bloecker K Wirth W Hudelmaier M Burgkart R Frobell R Eckstein F Morphometric differences between the medial and lateral meniscus in healthy men - a three-dimensional analysis using magnetic resonance imaging Cells Tissues Organs 2012 195 4 353 364 10.1159/000327012 21709397 PMC3696373

[B31] Jones RS Keene GC Learmonth DJ Bickerstaff D Nawana NS Costi JJ Pearcy MJ Direct measurement of hoop strains in the intact and torn human medial meniscus Clin Biomech 1996 11 5 295 300 10.1016/0268-0033(96)00003-4 11415635

[B32] Wenger A Wirth W Hudelmaier M Noebauer-Huhmann I Trattnig S Bloecker K Frobell RB Kwoh CK Eckstein F Englund M Meniscus body position, size, and shape in persons with and persons without radiographic knee osteoarthritis: quantitative analyses of knee magnetic resonance images from the osteoarthritis initiative Arthritis Rheumatism 2013 65 7 1804 1811 10.1002/art.37947 23529645

[B33] Jung KA Lee SC Hwang SH Yang KH Kim DH Sohn JH Song SJ Hunter DJ High frequency of meniscal hypertrophy in persons with advanced varus knee osteoarthritis Rheumatol Int 2010 30 10 1325 1333 10.1007/s00296-009-1153-7 19826824

[B34] Englund M Felson DT Guermazi A Roemer FW Wang K Crema MD Lynch JA Sharma L Segal NA Lewis CE Risk factors for medial meniscal pathology on knee MRI in older US adults: a multicentre prospective cohort study Ann Rheum Dis 2011 70 10 1733 1739 10.1136/ard.2011.150052 21646417 PMC4864962

[B35] Felson DT Nevitt MC Blinding images to sequence in osteoarthritis: evidence from other diseases Osteoarthr Cartil 2009 17 3 281 283 10.1016/j.joca.2008.09.008 18977156 PMC3653635

